# In vivo evaluation of a regenerative approach to nasal dorsum augmentation with a polycaprolactone-based implant

**DOI:** 10.1186/s40001-019-0364-y

**Published:** 2019-01-28

**Authors:** Paul S. Wiggenhauser, Elizabeth R. Balmayor, Nicole Rotter, Jan T. Schantz

**Affiliations:** 1 Department of Hand, Plastic and Aesthetic Surgery, Ludwig Maximilian University of Munich, Munich, Germany; 2 Department of Oto-Rhino-Laryngology, Head and Neck Surgery, Ulm University Medical Center, Ulm, Germany; 3 Experimental Trauma Surgery, Department of Trauma Surgery, Klinikum rechts der Isar, Technical University of Munich, Ismaninger Str. 22, 81675 Munich, Germany; 4 Department of Plastic Surgery and Hand Surgery, Klinikum rechts der Isar, Technical University of Munich, Munich, Germany; 5 School of Chemical and Biomedical Engineering, Nanyang Technological University, Singapore, Singapore

**Keywords:** Nasal dorsum augmentation, In vivo study, Polycaprolactone, Regenerative implant, Fibrous cartilage

## Abstract

**Background:**

Alternative techniques for nasal dorsum augmentation are of paramount importance in reconstructive and plastic surgery. In contrast to autologous cartilage grafts, tissue-engineered grafts can be created de novo and yield low–none donor site morbidity as compared to autologous grafts like rib or ear cartilage. To address this demand, this study investigated the in vivo regenerative potential of polycaprolactone-based implants as an alternative to autologous cartilage grafting during rhinoplasty.

**Methods:**

Implants were placed at the nasal dorsum in two groups of minipigs and kept in situ for 2 and 6 months, respectively. Subsequently, the implants were harvested and examined by histology (hematoxylin–eosin, alcian blue, and safranin O) and immunostaining (collagen I and collagen II). Further analysis was performed to measure diameter and distance of polycaprolactone struts.

**Results:**

Histological examination revealed a persistent formation of connective tissue with some spots resembling a cartilaginous-like matrix after 6 months. In such areas, cells of chondrocyte appearance could be identified. There was a significant decrease in strut diameter but a non-significant difference in strut distance.

**Conclusion:**

Our results indicated that the investigated polycaprolactone-based implants have shown a regenerative and stable nasal dorsum augmentation after 6 months in vivo. Thus, we believe that customized polycaprolactone-based implants could become an alternative technique for nasal dorsum augmentation without the need for autologous cartilage grafts.

## Introduction

The dorsum of the nose is a major esthetic key factor in rhinoplasty [1]. The size and shape of the nasal dorsum define the lateral and frontal profiles of the nose. Moreover, the nasion, i.e., the junction of the nasal bones and the forehead, influences the esthetic perception of the entire nose [2]. Therefore, rhinoplasty focuses not only on straightening the eyebrow tip line but also on remodeling the lateral profile, starting at the forehead down to the tip of the nose [1]. Therefore, osteotomy of the nasal bones and augmentative procedures, such as radix grafts, are routinely performed [3]. The gold standard for augmentation is the use of whole-cartilage grafts or cartilage-based grafts via the Turkish delight technique [3, 4]. Surgeons rely in some cases on artificial materials, such as Gore-Tex, with the risk of a foreign-body reaction or, more dramatically, extrusion through the skin [5]. Table 1 shows a comprehensive overview of various approaches in performing nasal dorsum augmentation.

**Table 1:** Approaches to nasal dorsum augmentation

Approach	Details	Advantages	Disadvantages	Refs.
Cartilage graft	Usage of different cartilage sources (septal, auricular, costal), handcrafted	Autologous material	Possible long-term deformation, partial resorption	[1, 36]
Turkish delight	Use of autologous cartilage and fascia	Partially autologous material, easily to produce and to form	Partial resorption and deformation, allograft (fascia)	[37, 38]
Bone graft	Mostly taken from rib (also as costal cartilage with adjacent rib) or calvarial bone	Stability, less warping, resembling bone–cartilage parts of the nose	Donor site morbidity, partially unnatural biomechanics	[39, 40]
Gore-Tex	Synthetic, sponge like materials	Easy to use, soft, shapeable, tissue ingrowth possible	Extrusion, foreign body reaction	[41, 42]
Polyethylene	Biomaterial with porous structure, e.g., Medpor	Low inflammatory reaction, ingrowth of surrounding tissue	Extrusion, infection, stiffness	[43]
Silicone implant	Preformed implant	Easy to use, cheap	Extrusion, dislocation, unnatural feeling, capsular formation, deformation of the nose	[44]
Fillers	Hyaluronic acid derivatives, calcium hydroxylapatite gel	Easy to use, resorbable (hyaluronic), long-term stable (hydroxylapatite), easy dosing	Infection, necrosis, thinning of skin	[45, 46]
Fat transplantation	Autologous alternative to the use of dermal fillers, exploiting lipofilling technologies	Autologous material, easy to dose, soft, repeatable procedure	Large volumes need multiple procedures, larger volumes lacking stability and persistence of shape	[47]

In our opinion, there is an alternative to the common techniques used for nasal dorsum augmentation. In an earlier study, we showed a safe and easy use of tissue engineering-derived surgical implants in maxillofacial surgery [6, 7]. This technology is based on computer-assisted design/computer-assisted manufacturing (CAD/CAM) and polycaprolactone (PCL) implants for the regeneration of bone [8–12] and other tissues [13, 14]. PCL is a biocompatible material and PCL-based implants biodegrade within 2 years and promote autologous bone formation [15]. This is a stable and sustainable technique to augment the bony parts of the nasal dorsum and forehead. On the contrary, cartilage grafts are often completely or partially resorbed, sometimes with unpredictable, uneven outcomes and the need for another surgery [16].

Based on the previously acquired in vitro and in vivo data, this study was designed to evaluate the potential use of PCL-based implants for nasal dorsum augmentation in clinical routine because of their ability to induce connective tissue regeneration.

## Materials and methods

First, this study evaluated the potential of PCL-based implants with regard to cartilage regeneration. Therefore, nasal dorsum augmentation was performed using newly designed implants in six animals. The implants were explanted after 2 months (group 1; *n* = 3) and 6 months (group 2; *n* = 3) and examined histologically, with at least three representative images taken for each. The final examination focused on the proof of cartilage regeneration and the stability of the implants over time.

Second, the study aimed at a potential preclinical evaluation of PCL-based implants. Therefore, implant manufacturing, surgical procedures, and animal keeping were conducted in conformance with good manufacturing practices (GMPs) and with the approval of the local ethics committee (PN13004). An Association for Assessment and Accreditation of Laboratory Animal Care International (AAALAC)-accredited GMP facility (PWG Laboratories, Singapore) was used, which had a specific pathogen-reduced large-animal area, state-of-the-art operation theaters, and an in-house diagnostic center.

**Fig. 1:**
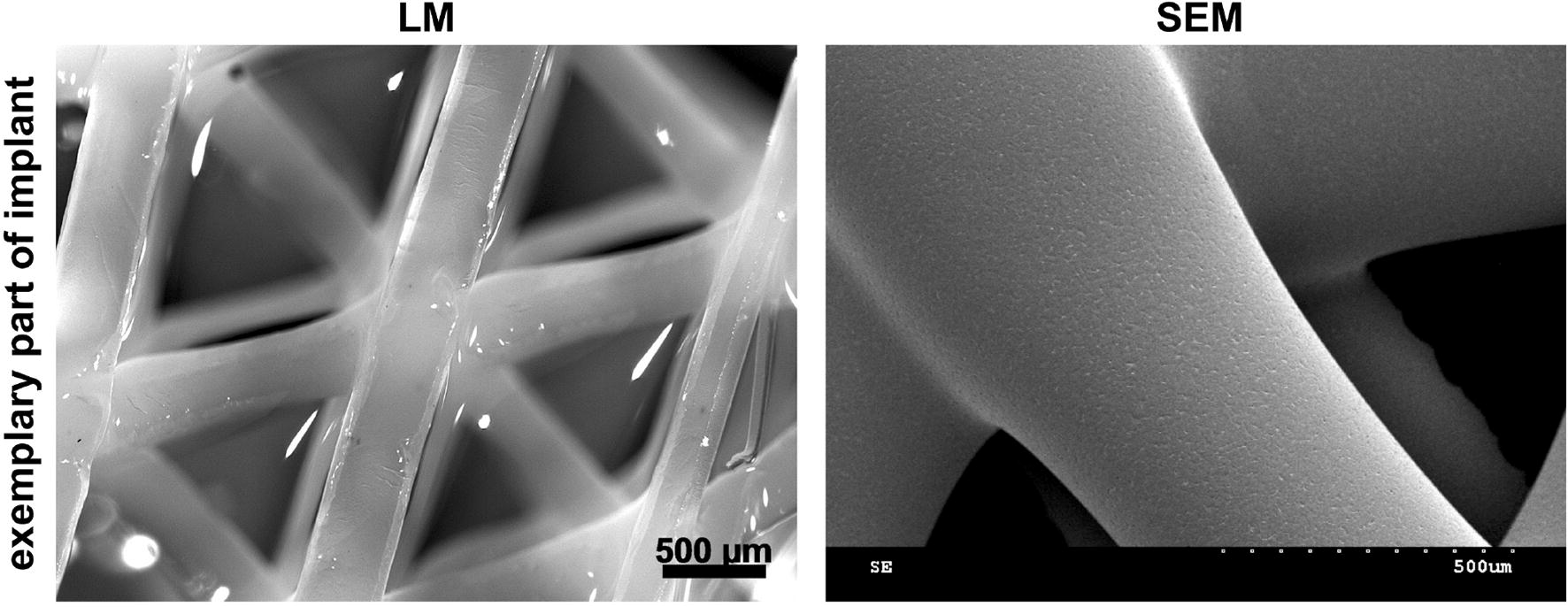
Exemplary parts of scaffolds are demonstrated by light microscopy (LM) and scanning electron microscopy (SEM)

**Fig. 2:**
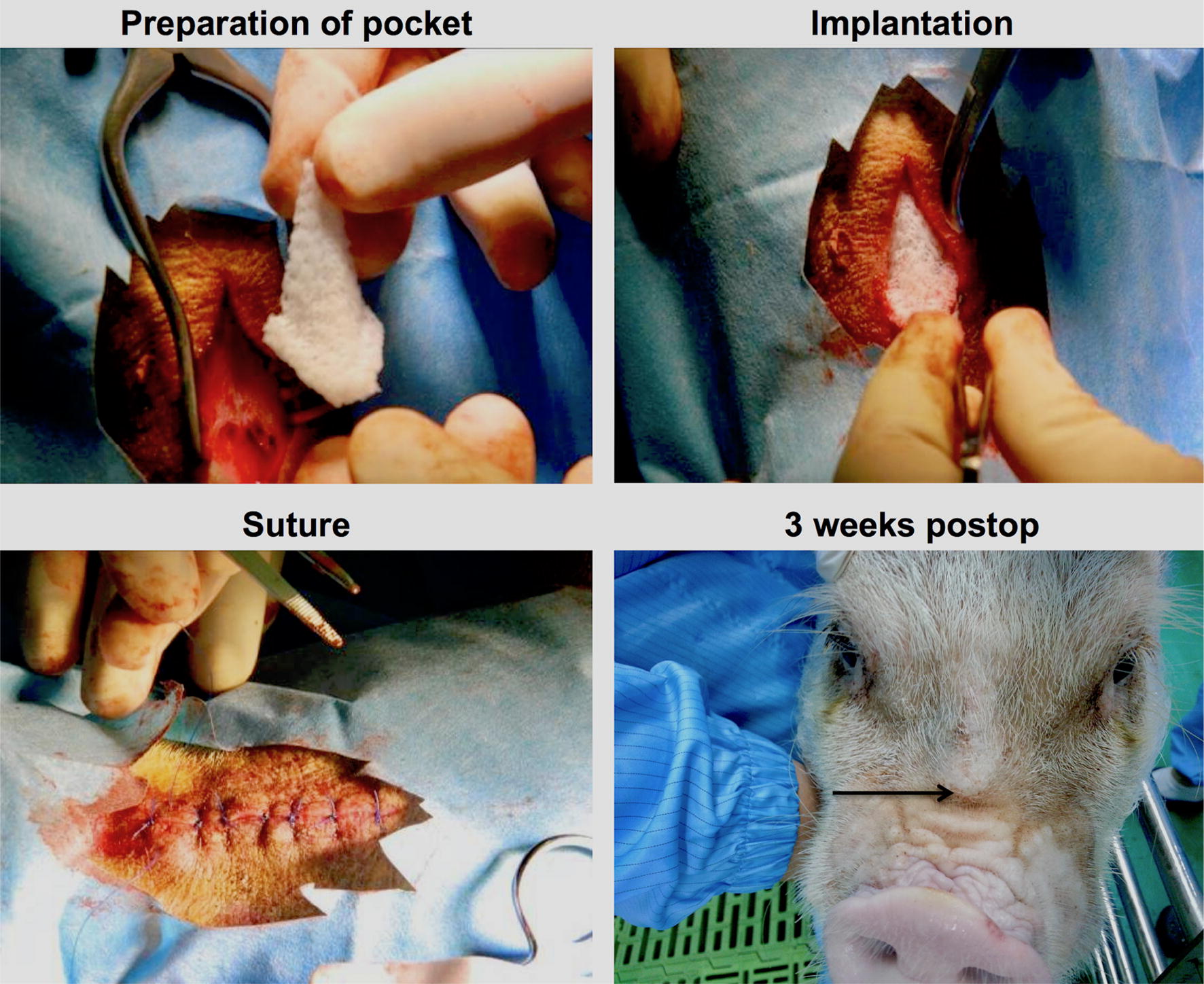
Nose scaffold was manually trimmed and then implanted just above the snout where there is a concave deepening. An incision was made deep into the thick fibrous tissue above the periosteum, fibrous tissue was cut to loosen the skin around the implant, and the skin was then sealed using sutures

Nasal implants were manufactured and supplied by Osteopore International Pte. Ltd. (Singapore) using 3D printing technology. Medical grade PCL (mPCL, molecular mass (Mn) of 80 kD, Osteopore) was used. The implant shape was defined with the desired geometry using CAD. PCL was melted and then extruded in a predetermined lay-down pattern, also known as fused deposition modeling. Exemplary parts of the implant are demonstrated in Fig. 1 to illustrate the internal 3D structure of the implant and to visualize the smooth surface of the material. Biomechanical properties of comparable PCL-based implants fabricated by 3D printing for cartilage regeneration have been reported elsewhere [17, 18]. The manufacturing process was conducted in a clean-room environment (ISO 14644 compliant). All implants were sterilized with gamma irradiation according to ISO 11137 guidelines. The implants were released for use after undergoing strict quality control (under the ISO 13485-compliant quality management system of Osteopore), which included maintaining a porosity of 70% ± 5%. The implants were pyramid like with the perpendicular height taller than the length of the triangular base, as seen in Fig. 2 (preparation of pocket). The height was 10 cm and the length of each base edge was 1.5 cm. During surgery, the implant was shortened manually by cutting the top and base edges of the pyramid to fit the implant snugly into the defect space that was created, allowing intimate contact with the host tissue, as depicted in Fig. 2 (implantation).

To underline the preclinical aspect of this study, mature female miniature pigs (minipigs) (PWG Laboratories, Singapore) were used for in vivo investigation. They were sedated with ketamine 10 mg/kg and xylazine 2–5 mg/kg, which was administered intramuscularly approximately 10–20 min before induction. Each animal was masked with isoflurane at 5% before intubation, and anesthesia was maintained at 2–5% isoflurane. After meticulous irrigation and shaving of the dorsum, a 5-cm longitudinal incision was made and a subcutaneous pocket was prepared over the nasal periosteum. Subsequently, the implant was inserted into the pocket after careful hemostasis. The implant was consecutively positioned over the bony aspects of the nose avoiding the cartilagineous parts of the nose. The wound was closed with 2.0 Ethilon sutures. The animals were postoperatively administered 1 mL/10 kg Betamox twice every 48 h, extended to four times, if necessary. Pain relief was achieved with 1 mL/25–50 kg meloxicam once daily for 2 days, extended to 4 days, if necessary. In addition, 2–4 mg/kg tramadol was added once up to twice daily, if required.

There were no significant adverse side effects of treatment, no dislocation, and no significant infection in any animal. Specimens were harvested under general anesthesia (described before) 2 and 6 months (groups 1 and 2, respectively) after initial surgery. The former incision was reopened with a scalpel, and the subcutaneous tissue was dissected. The implant was sharply dissected out of the surrounding tissue and instantly fixed in 3.7% neutral buffered formalin solution for at least 24 h.

The specimens were dehydrated using gradually increasing concentrations of ethanol and embedded in paraffin. Using a rotary microtome (HM 340 E, Zeiss), 5-µm thick sections were made. Hematoxylin–eosin (HE), alcian blue (AB), and safranin O staining were performed following standard protocols. The slides were observed and photographed with an inverted microscope (Biorevo BZ9000, Keyence, Osaka, Japan) at various magnifications. A general picture of the entire histological section was obtained using the software BZ-II Viewer and BZ-II Analyzer (Keyence).

Immunohistochemical detection of collagen I and II was achieved using a peroxidase-based labeling system (SAB0+ System-HRP, DAKO, Glostrup, Denmark) as described elsewhere [19]. In brief, the specimens were deparaffinized, and the rehydrated sections were each treated with 1% hyaluronidase (Sigma-Aldrich, St. Louis, MO, USA) in phosphate-buffered saline (PBS) and 0.2% pronase (Merck, Darmstadt, Germany) in PBS for 15 min at 37 °C. For collagen II staining, the Elsaesser procedure was followed using collagen II antibody (II-II6B3, Developmental Studies, Hybridoma Bank, USA) diluted at a ratio of 1:1000 and incubated for 1 h at room temperature [20]. For collagen I staining, rehydrated specimens were treated with 1 mg/mL pepsin in 0.5 M acetic acid solution for 2 h at 37 °C. The endogenous peroxidase was blocked twice with a serum-free protein block (DAKO) for 30 min. The primary antibody (ab34710, Abcam, United Kingdom) was diluted at a ratio of 1:4000, incubated on the sections at 4 °C overnight, and visualized analogously to the collagen II specimens with SAB+ System-HRP. All sections were counterstained with hematoxylin and mounted with RotiHistokit II (Carl Roth, Germany) before image acquisition. Positive and negative controls were made with porcine septal cartilage and adjacent connective tissue samples.

Advanced image analysis was conducted with three measurements of each specimen, and values were averaged for each animal (*n *= 3). First, cross sections of PCL scaffold struts were identified in HE-stained specimens as round or oval voids within the histological sections. These voids formed due to the melting of the struts during paraffin embedding of the samples, leading to a footprint-like negative image of the scaffolds. Next, the maximal diameter of each identified strut was measured, and the distance between the centers of two parallel but not directly adjacent struts (strut k and strut k + 2) was calculated to evaluate the persistence of space between the different layers of the fused deposition modeling.

Unpaired, two-tailed Student’s *t* tests were used for statistical analysis in IBM SPSS Statistics 21 (IBM Corp, USA), and graphs were created using Microsoft Excel 2013 (Microsoft, USA).

## Results

CAD/CAM technology allowed flexible modeling of the implant shape and manufacturing process, and the implants were successfully manufactured using 3D printing in the previously defined lay-down pattern. Because of their predefined geometrics, less effort was needed to adopt the implants to the surgical implant location.

The animals tolerated the procedure well. There were no signs of a clinically detectable foreign-body reaction or infection. Immediately after the procedure, a significant augmentation effect was observed, which was present throughout the entire study. No implant dislocation or fracture was observed. On manual palpation, the implants were firm and covered by a thick soft-tissue layer. All implants showed sufficient integration in the surrounding tissue, without any signs of capsular formation.

**Fig. 3:**
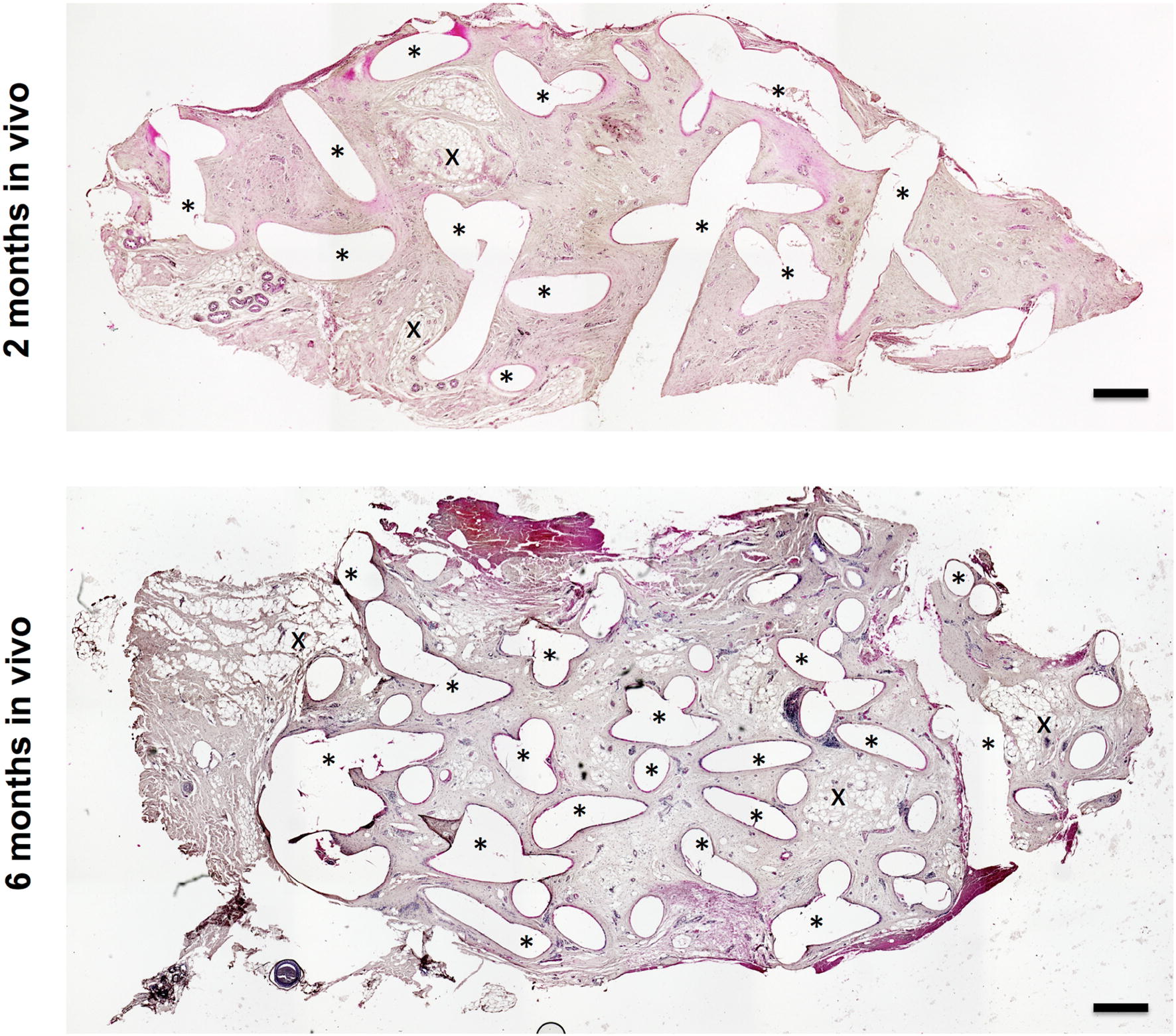
Cross sections of entire implants stained with hematoxylin–eosin. Polycaprolactone melts during paraffin embedding. The resulting artifacts, i.e., empty voids, are marked with asterisks (*). Spontaneous fat tissue formation is indicated with an X (scale bar = 1 mm)

**Fig. 4:**
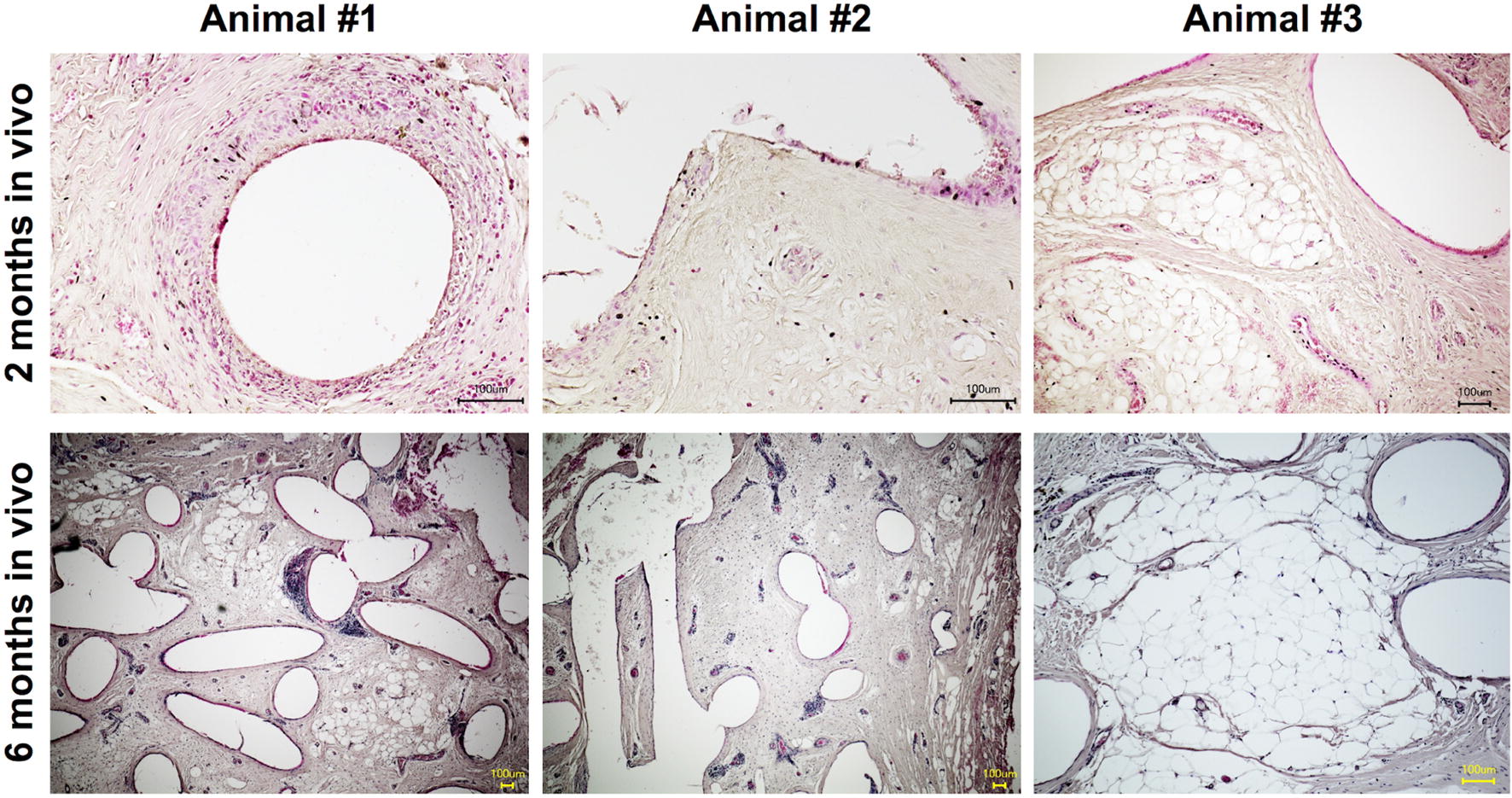
Cross sections of implants and exemplary findings of all specimens stained with hematoxylin–eosin. Few spots show a foreign-body reaction to polycaprolactone. Most parts of the implant are covered with a thin cell lining, demonstrating good integration and high tolerability by the host tissue (scale bar = 100 µm)

**Fig. 5:**
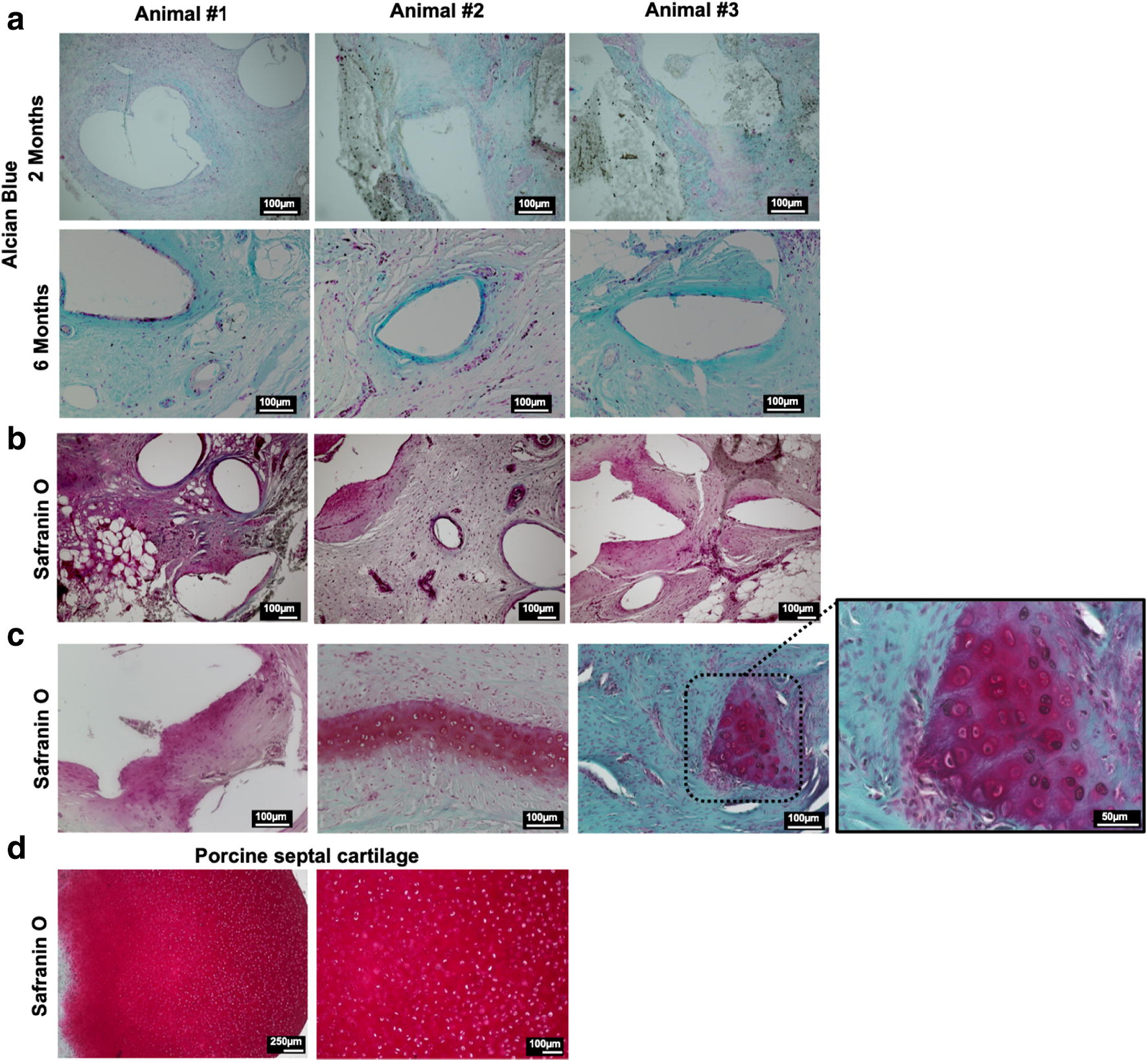
Representative histology images for alcian blue (AB) and safranin O staining for all three animals (*n* = 3) after 6 months in vivo. **a** AB staining for the scaffolds harvested 2- and 6-months post-implantation. The images clearly show intense blue staining around the polycaprolactone fibrils. **b** Staining with safranin O confirms the presence of a cartilaginous-like matrix (bright-red color). **c** Higher magnification images show morphological characteristics of the cells populating the new tissue formed within the implants. **d** Histological images of porcine septal cartilage as comparison to experimental groups

HE staining (Figs. 3, 4) revealed an ingrowth of cells within the entire implants, with preferential distribution around the scaffold fibers at the interface between tissue and scaffold. There was no unexpected allocation of inflammatory cells (as defined by HE morphology) in any of the samples. Furthermore, capillary, arterial, and venous structures were observed within the scaffolds indicating the presence of vascularization. AB staining (Fig. 5a) was performed to identify acidic polysaccharides, such as glycosaminoglycans, in the collected implants. Scaffolds harvested after 2 months showed clear formation of connective tissue that was well populated by cells. Furthermore, scaffolds harvested after 6 months showed persistent cartilaginous-like matrix around the PCL scaffold struts. Here as well, abundant connective tissue was observed being replaced by the cartilaginous matrix in closer proximity to the scaffold implant. The areas of connective tissue and cartilaginous-like matrix formation, as identified in AB staining, were clearly localized in the vicinity of the struts. These results were confirmed by safranin O staining. All samples showed positive red matrix staining, indicating cartilaginous-like tissue in areas previously described and identified with AB staining.

**Fig. 6:**
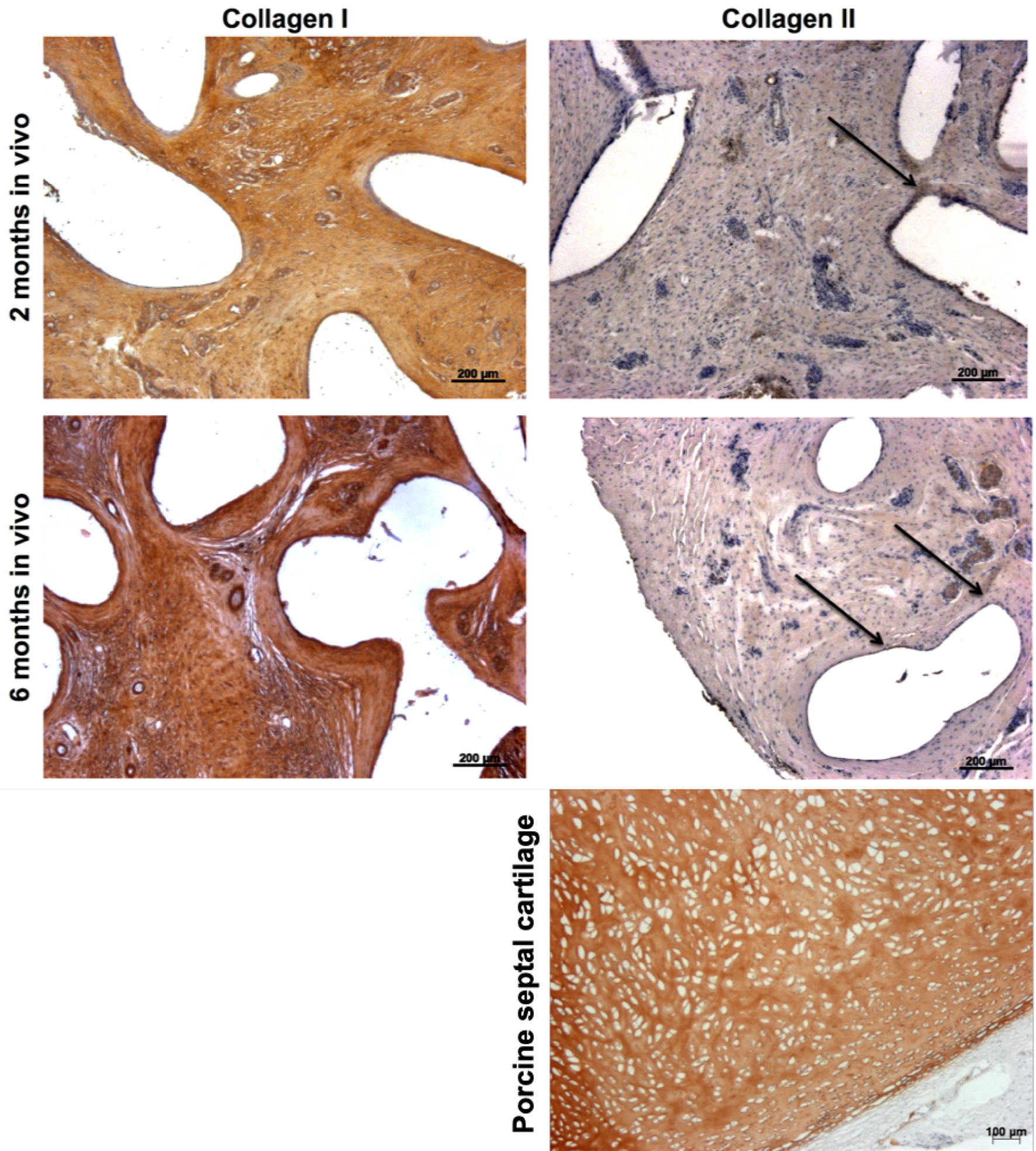
Immunohistology confirms the abundant presence of collagen I. An increase on staining intensity could be observed over time. On the contrary, collagen II shows only a faint staining (as indicated by arrows). Representative images of the harvested samples are depicted. An image of porcine septal cartilage is depicted as comparison to experimental groups

To investigate the tissue type and differentiation potential, collagen I and II immunostaining was performed. The results are shown in Fig. 6. Collagen I staining was positive in all samples. There was a visible increase of collagen I over time, particularly significant in the inner parts of the scaffolds and the tissue directly adherent to the PCL fibrils. However, collagen II was hardly detected in any sample; few samples showed a faint reaction after 6 months in vivo.

**Fig. 7:**
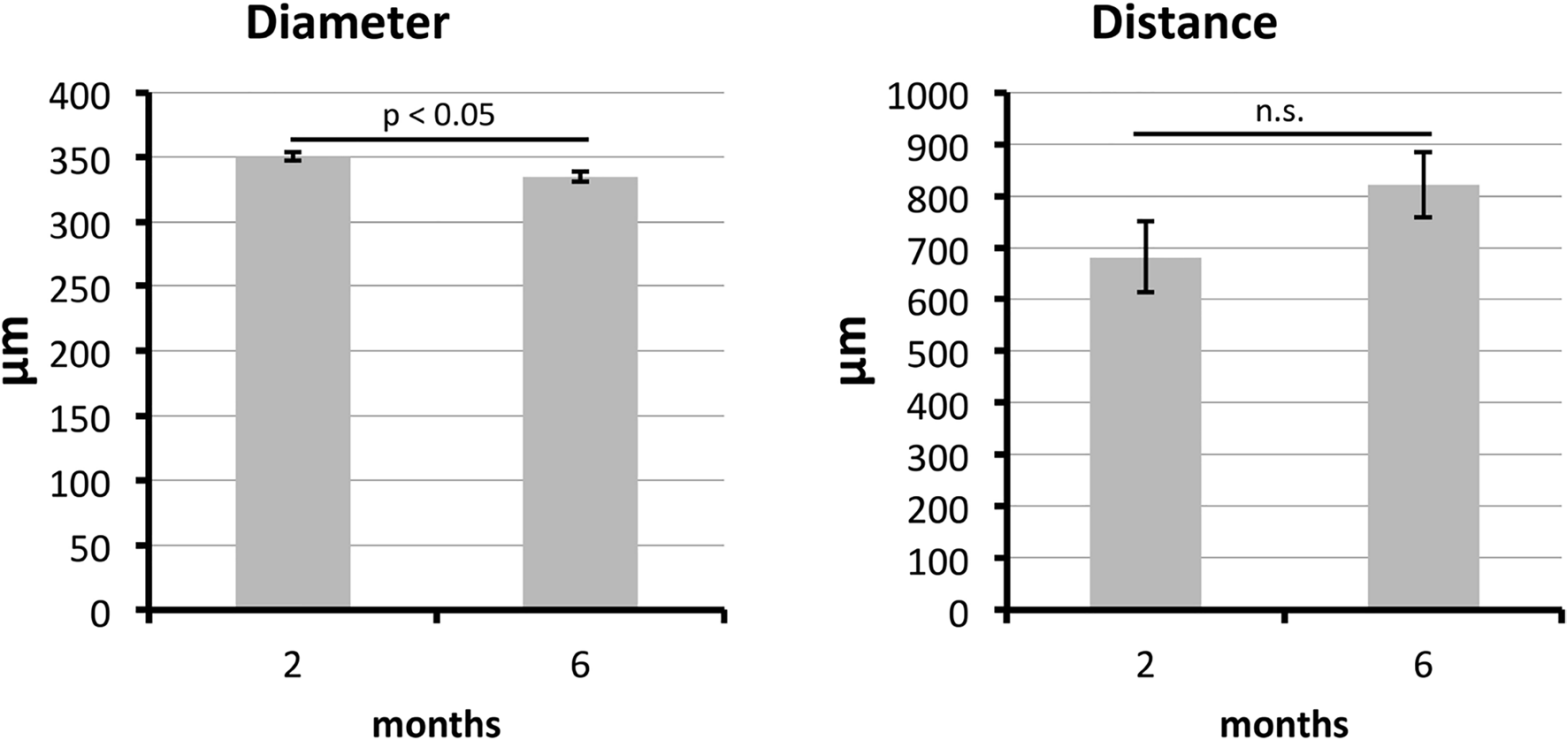
Results of the image analysis regarding implant geometry depicted as bar plots with standard deviation. The polycaprolactone scaffold strut diameter and the distance between the central axes of the struts are shown

Image analysis revealed that the average diameter of the struts was 350 ± 3 µm after 2 months and 335 ± 4 µm after 6 months; there was a statistically significant difference in these values. The distance between the central axes of the struts was 682 ± 69 µm after 2 months and 821 ± 63 µm after 6 months; the difference was not statistically significant, and values were approximately within the desired range, as calculated (expected distance = 2 × diameter of strut) (Fig. 7).

## Discussion

This study investigated the suitability of PCL-based implants for nasal dorsum augmentation. Compared to other tissue engineering-based studies, our study used neither scaffolds with bioactivation nor precultivation of stem cells in vitro. Instead, scaffolds were implanted analogously to conventional surgical implants at the nasal dorsum to demonstrate the regenerative potential of the implants themselves without the compromising side effects of additional factors added to the implants.

The literature describes two main regenerative approaches: (i) to recreate the underlying structural tissues with cartilage grafts and (ii) to expand the subcutaneous soft tissue. The latter is based on clinically available matrices (i.e., Integra and Alloderm) for dermal regeneration [21, 22]. These matrices are mainly collagen based and have shown regenerative potential for connective tissue. Their advantages are their softness, which allows smooth texturing of the nasal dorsum, and their applicability in clinical routine as both materials have regulatory approval [21]. However, the nature of the materials is such that they cannot act as functional and stabilizing substitutes for the nasal dorsum itself [22]. Therefore, their application is reduced to only smoothing and augmentation of the nasal dorsum. On the contrary, tissue engineering-based approaches with autologous chondrocytes cultivated on a cell carrier to shape the desired implant allow the generation of a functional substitute of the nasal dorsum [23]. Yanaga et al. used chondrocyte injections to generate vivid cartilage in vivo and to augment the nasal dorsum and tip in more than 75 patients [24]. In vitro expansion of the chondrocytes obtained from the auricular cartilage was necessary to offer a suitable cell number for the desired volume effect [24, 25]. Kim et al. used 3D-printed PCL scaffolds to generate a long-lasting volume effect of the desired geometry in nasal dorsum augmentation in a preclinical animal model. However, there was no tissue regeneration observed, particularly no cartilage formation, after 3 months in vivo [26]. The time of observation selected by the authors, i.e., 3 months, might have been insufficient for complete cartilage regeneration to be observed.

In contrast, several in vitro and in vivo studies have demonstrated the osteogenic induction of PCL-based implants [7, 15, 27]. However, less information is available about the fate of such implants in the case of subcutaneous implantation without the addition of further bioactive factors. Chanchareonsook et al. investigated the tissue reaction of New Zealand rabbits to PCL-based implants in comparison to titanium implants [28]. They found similar tissue adhesion, a thinner fibrous capsule, and a higher number of inflammatory cells in the PCL group [28].

Our study demonstrated the potential of PCL-based implants in reference to cartilage tissue regeneration. Histology and immunohistology examinations showed the presence of cartilaginous-like tissue rich in cartilage-defining proteins after 6 months of implantation. Unfortunately, the low ratio of collagen II may indicate that there was more fibrous cartilaginous-like tissue than mature hyaline cartilage. Compared to Kim et al.’s study [26], our study showed the formation of a tissue with cartilage-like characteristics that may progress to mature cartilage tissue over time. Why the combination of PCL, fibrin glue, and chondrocytes, as previously used by Kim et al., did not show sufficient chondrogenesis remains unclear, considering that several studies have demonstrated the efficacy of the method [29]. This may be because of the longer observation time in our study or the exploitation of in situ regeneration by strict perichondral/periosteal implantation of unseeded scaffolds.

Compared to nasal dorsum augmentation with autografts, PCL-based implants yield a stable augmentation effect with almost none of the unwanted changes of contour. In contrast, allografts with solid or minced cartilage can be resorbed partially or totally within months after surgery. This effect may be due to cartilage atrophy after transplantation [30]. In our study, all group 2 animals showed constant augmentation of the nasal dorsum after 6 months in vivo. Further research will be necessary to determine long-term stability after 1 and 2 years in vivo.

Theoretically, the long-term stability of PCL-based implants is based on the controllable degradation time of PCL. The hydrolytic bioresorption of PCL occurs by cleavage of the ester linkages within the polymer in two phases [8, 31–33].

Phase 1 involves the nonenzymatic hydrolytic cleavage of the ester linkages, leading to bulk degradation of PCL. The mass and volume, and therefore the shape, of the implant remain unchanged [31–33].

This phase shows a controlled, predictable, first-order, linear bioresorption pattern; therefore, an increase in the initial molecular weight results in a longer resorption time. Pitt et al. found a degradation time of 112 weeks for PCL with a molecular weight of 51,000 Da. By this means, the degradation time of PCL-based implants can be controlled by the initial molecular weight of the PCL used for implant production [31–33].

Phase 2 starts at a specific chain length that has been determined to correspond to a chain molecular weight of approximately 3000–5000 Da, allowing the small fragments to diffuse through the polymeric matrix. This phase is characterized by (i) the onset of controlled and predictable total mass loss via bioresorption of the microspheres and excretion through the normal metabolic pathways and (ii) a decrease in the rate of chain scission [32, 34].

These observations in the literature correspond well with our findings of a significantly reduced diameter of the PCL scaffold struts but not of the significantly altered distance between them, indicating a still stable scaffold architecture and volume. Taking these arguments into consideration, we hypothesize that stability of PCL-based implants is given and that controlled augmentation of the nasal dorsum could be achieved using such implants. But further studies with longer in vivo periods will be necessary for preclinical evaluation.

Current surgical techniques are based on autologous materials like cartilage or allografts like silicone-based implants. However, both kinds of implants yield different aspects of clinical risks: foreign-body reaction, extrusion, and total/partial resorption [4]. PCL, on the other hand, has multiple advantages with regard to the presented application:

- PCL has been shown to have low foreign-body reaction [28] and long-term stability in vivo with degradation times over 2 years [8]. With regard to further clinical studies, we found excellent tolerance of procedures with none–few unwanted side effects. No implant showed signs of extrusion or dislocation.
- PCL-based implants have been used for over 10 years in clinical regenerative applications. There are several case reports found in the literature. For example, Schuckert et al. showed the possibility of reconstructing a critical bone defect of the mandible using a PCL-based implant in a 71-year-old female patient [6, 35].
- PCL-based implants are approved by the US Food and Drug Administration (FDA) for craniofacial applications. Therefore, the implants can be used in clinical practice directly because they are derivatives of existing craniofacial implants. This makes PCL-based implants a sustainable alternative for nasal dorsum augmentation.

The underlying CAD/CAM technology allows further improvement of the implants in many ways. First, the geometry and overall design of the implant can be easily adjusted in size according to the patient’s requirement before production. Second, a fully customized implant can be produced and fitted to the patient’s face using cone beam computed tomography scans and specialized algorithms for automatized CAD modeling. Moreover, further modifications may address the porosity of the implant or the geometry of the lay-down pattern. Further research could also address bioactivation to improve cartilage regeneration and to generate hyaline cartilage instead of fibrous cartilage or cartilaginous-like matrix.

In summary, PCL-based implants have shown a tolerable implant design and high regenerative potential in vivo. Moreover, existing long-term results from other studies, including first-in-human trials, have supported the findings of our study. Therefore, nasal dorsum augmentation with regenerative implants might be considered a new alternative to conventional surgical techniques. This technology could offer surgeons a new way to address their patients’ expectations in plastic surgery procedures. But further research will be necessary to prepare clinical studies.

## Conclusion

This study is pioneer in reporting a preclinical evaluation of PCL-based implants for nasal dorsum augmentation. We used minipigs and a clinically relevant model to assess the potentialities of PCL implants. FDA approval for specific PCL-based maxillofacial implants exists. Therefore, PCL-based implants could become an interesting alternative for patients seeking a regenerative approach to surgery. However, further research is required to evaluate long-term stability and effectiveness in humans.
